# Ensuring the Reliability of Virtual Sensors Based on Artificial Intelligence within Vehicle Dynamics Control Systems

**DOI:** 10.3390/s22093513

**Published:** 2022-05-05

**Authors:** Philipp Maximilian Sieberg, Dieter Schramm

**Affiliations:** Chair of Mechatronics, Faculty of Engineering, University of Duisburg-Essen, 47051 Duisburg, Germany; dieter.schramm@uni-due.de

**Keywords:** artificial intelligence, artificial neural network, control systems, hybrid state estimation, reliability, vehicle dynamics

## Abstract

The use of virtual sensors in vehicles represents a cost-effective alternative to the installation of physical hardware. In addition to physical models resulting from theoretical modeling, artificial intelligence and machine learning approaches are increasingly used, which incorporate experimental modeling. Due to the resulting black-box characteristics, virtual sensors based on artificial intelligence are not fully reliable, which can have fatal consequences in safety-critical applications. Therefore, a hybrid method is presented that safeguards the reliability of artificial intelligence-based estimations. The application example is the state estimation of the vehicle roll angle. The state estimation is coupled with a central predictive vehicle dynamics control. The implementation and validation is performed by a co-simulation between IPG CarMaker and MATLAB/Simulink. By using the hybrid method, unreliable estimations by the artificial intelligence-based model resulting from erroneous input signals are detected and handled. Thus, a valid and reliable state estimate is available throughout.

## 1. Introduction

Active safety systems are a key factor in achieving the objective of no accidents incorporating vehicles, as addressed by [1]. These active safety systems include driver assistance systems and vehicle dynamics control systems. Driver assistance systems address the driving task of guidance, in contrast vehicle dynamics control systems operate at the level of stabilization [2]. Both types of systems require information about the environment and the states of the vehicle dynamics. One possibility to obtain the necessary knowledge is the direct acquisition by sensors. Virtual sensors are additionally used especially in the field of vehicle dynamics control. These are based on mathematical models that determine the required states on the basis of already known quantities.

In addition to the classical theoretical modeling approach, it is also possible to generate these mathematical models through experimental modeling. Machine learning methods and artificial intelligence in general are frequently used for this purpose [3]. The application of physical-based models within virtual sensors, for example by using Kalman filters, is a well-researched field. In [4], a Kalman filter is utilized to predict the roll and pitch angle. By integrating a second measurement update, the estimation accuracy is enhanced. Ref. [5] compare the performance of an extended and an unscented Kalman filter for vehicle dynamics state estimation. The unscented Kalman filter outperforms the extended Kalman filter, especially due to linearization errors at larger sampling times. All approaches share one superior requirement in common. The determination of the states must be reliable and valid. If not, the safety is compromised especially in connection with vehicle dynamics control systems. This also applies to models based on artificial intelligence. These have the potential to improve the accuracy of the estimation significantly while reducing the modeling effort [6,7]. In [8] for example, an artificial neural network is used to predict the roll angle, in order to be able to realize a vehicle roll control system. Ref. [9] apply a deep learning approach to simultaneously estimate the roll angle and the side-slip angle of a vehicle. However, most artificial intelligence-based models, such as artificial neural networks, belong to the class of black-box models, whose modes of operation are not fully comprehensible and therefore not reliable [10]. 

In addition to purely physical and artificial intelligence-based estimators, respectively, combinations of both approaches can be utilized. In [11], a hybrid method is used, where an unscented Kalman filter accounts for a pseudo-measurement derived by an artificial neural network. From this integration, the noise within the estimation can be reduced and the estimation quality is enhanced. Similar results are achieved by [12], where an observer is used instead of a Kalman filter. A different hybrid method is employed by [13], where a pseudo-measurement quantity derived by a physical model is used as an input into an artificial neural network. Using this integration, the estimation accuracy of the artificial neural network is improved. However, the focus of these hybrid methods is on increasing the accuracy and not on ensuring the reliability. 

This issue has been addressed and overcome by [14] using a hybrid method of state estimation. By combining and safeguarding an artificial neural network with a reliable physical model, a valid and reliable state estimation is obtained throughout. The roll angle estimation of a passive vehicle has been used as an application example. 

Within this contribution, the hybrid method of state estimation is adapted and validated together with a central predictive vehicle dynamics control [15]. The application example of the state estimation remains the roll angle estimation, as a possible rollover is a particularly large hazard for the vehicle occupants.

The paper is structured as follows: Section 2 introduces the simulation framework used to implement and validate the hybrid method of state estimation. Section 3 presents the hybrid method of state estimation comprising the physical model and the artificial intelligence-based model used. The validation of the reliability of the hybrid method of state estimation is given in Section 4. The paper concludes in Section 5 with a summary as well as an outlook on future research tasks.

## 2. Simulation Framework

**Figure 1:**
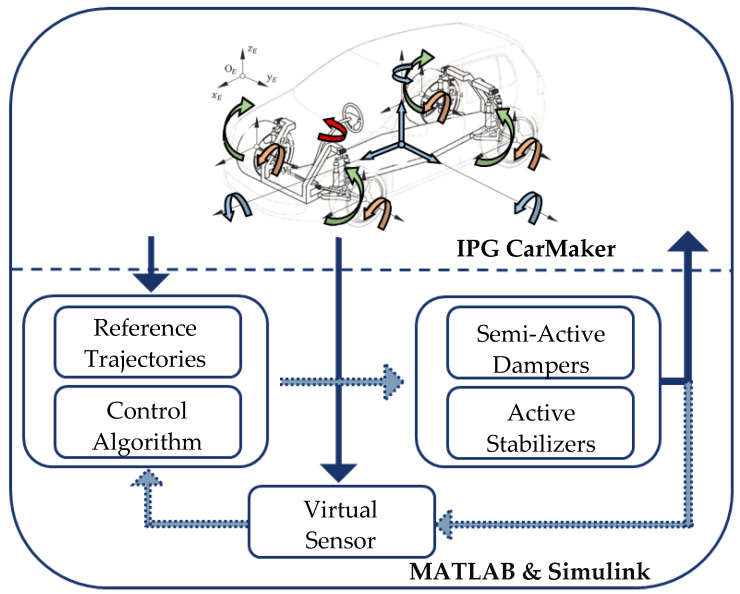
Simulation Framework, cf. [15].

To implement and validate the hybrid method of state estimation, a simulation framework is utilized. This simulation framework is shown in Figure 1.

The simulation framework is based on a co-simulation between IPG CarMaker and MATLAB/Simulink. IPG CarMaker represents the vehicle by a multi-body simulation. Furthermore, test tracks and driver models can also be adapted in IPG CarMaker.

MATLAB/Simulink is used for the central predictive vehicle dynamics control including the generation of reference trajectories, the simulation of actuator models and the state estimation itself. Here, the vehicle is equipped with two active stabilizers and four semi-active dampers.

The focus of this contribution is on the task of vehicle dynamics state estimation.

## 3. Hybrid Method of State Estimation

**Figure 2:**
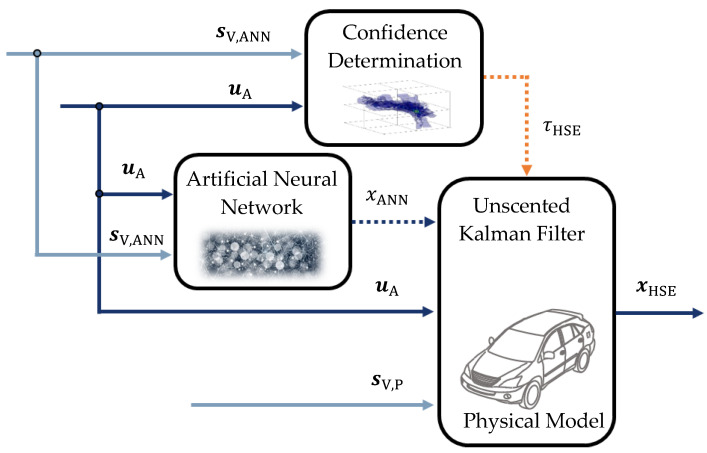
Hybrid Method of State Estimation—Structure, according to [16].

The objective of the hybrid method of state estimation is to increase the estimation quality compared to conventional physical virtual sensors and to ensure a reliable mode of operation. Virtual sensors resulting from experimental modeling, such as artificial neural networks, have the potential to increase the estimation quality. Unfortunately, the black-box characteristic they exhibit results in state estimations that are not fully secure and reliable. This issue is solved by the hybrid method of state estimation. Figure 2 illustrates the structure of the hybrid method.

In the hybrid method of state estimation, a physical model safeguards the artificial neural network. The hybrid method consists of three steps:

In the first step, the state is estimated by the artificial neural network on the basis of the actuating variables $u_{A}$ and measured variables $s_{V,\mathrm{ANN}}$. This results in the state $x_{\mathrm{ANN}}$ estimated by the artificial neural network.

In the second step, the input data into the artificial neural network are used to quantify the confidence in its estimation. This is achieved by determining a confidence level $\tau_{\mathrm{HSE}}$ [16].

In the third and final step, the state estimation $x_{\mathrm{ANN}}$ of the artificial neural network is combined with a reliable physical model as a function of the confidence level and thereby safeguarded. An unscented Kalman filter realizes the combination. This ultimately results in a hybrid state estimation $x_{\mathrm{HSE}}$.

Regarding the functionality of the hybrid method, the used measurement quantities are different for the artificial intelligence-based model with $s_{V,\mathrm{ANN}}$ and for the physical model $s_{V,P}$.

### 3.1. Artificial Neural Network

In order to implement an artificial neural network with an excellent estimation quality, first a hyperparameter optimization is performed. The driving maneuvers simulated in [16] are used as the database for hyperparameter optimization as well as training. During this hyperparameter optimization the parameters of the artificial neural network are adjusted and optimized, which remain unchanged during the actual training. Sequential model-based global optimization is used as the optimization approach [17]. The acquisition function within this informed optimization is based on the expected improvement [18]. In principle, a recurrent neural network based on long short-term memory cells is used for the estimation of the roll behavior, which is particularly suitable for mapping temporal relationships [19].

During the hyperparameter optimization, some basic parameters are kept fixed. These include the batch size, the number of training epochs and the utilized optimizer. Table 1 summarizes the fixed parameters.

Within the optimization, among other things, the hyperparameters of the number of recurrent layers, the number of neurons within the recurrent layers, the regularization and the temporal lookback are adapted [16]. The results of the hyperparameter optimization are shown in Table 2.

Furthermore, the input data into the artificial neural network are composed of the actuated manipulated variables $u_{A}$ and the measured variables $s_{V,\mathrm{ANN}}$. The measured input quanitities $s_{V,\mathrm{ANN}}$ into the artificial neural network are composed by the steering wheel angle δ, the velocity v and the yaw rate $\dot{\psi }$:

(1) $$s_{V,\mathrm{ANN}}={\left[\delta ,v,\dot{\psi }\right]}^{T}$$

The final training is conducted for 100 epochs with a learning rate of 0.00245. During the training, the options “reduce learning rate on plateau” and “early stopping” are activated [20,21]. The recurrent layers are followed by a dense layer, in order to retrieve the output quantity $x_{\mathrm{ANN}}$.

### 3.2. Confidence Level Determination

In a second step, the confidence in the estimation by the artificial neural network is quantified by determining a confidence level $\tau_{\mathrm{HSE}}$. To determine the confidence level, the input data used in the training of the artificial neural network are first classified into a $k_{c}^{n}$ dimensional grid structure. The n input variables are sorted into $k_{c}$ individual segments over the entire input space. Thus, each cell in the grid structure represents a certain part of the training of the artificial neural network. The number of data points indicates how extensively the training has taken place in this part. A larger number of data points correlates with a more extensive training and a better expected performance of the artificial neural network for the specific area [22].

During operation of the hybrid method, the latest input data into the artificial neural network are then used to determine the specific cell describing the actual vehicle dynamics situation. The number of data points $p_{c}$ within this specific cell is used to describe the extent of the training. Here, a larger number of data points also correlates with a higher confidence level. The number of data points $p_{c}$ is then scaled by the maximum number of data points $p_{\max}$ in a cell across the entire grid structure. This results in a confidence level $\tau_{\mathrm{HSE}}$ between zero and one:

(2) $$\tau_{\mathrm{HSE}}=\frac{p_{c}}{p_{\max}\;}$$

### 3.3. Physical Model

**Figure 3:**
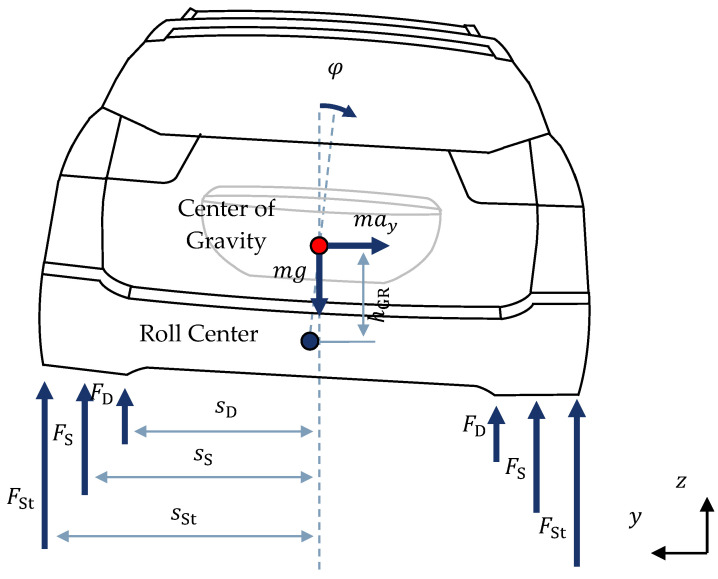
Free Cut of the Vehicle Body in the y-z Plane, cf. [16].

The artificial neural network is backed up within the hybrid method by a comprehensible physical model resulting from theoretical modeling. For the description of the roll behavior, a non-linear roll model is therefore derived according to [16]. The free cut is shown in Figure 3. 

The free cut of the vehicle body and setting up of the principle of angular momentum around the roll axis provides:

(3) $$\begin{array}{l} h_{\mathrm{GR}}ma_{y}\left(k\right)\cos\varphi_{P}\left(k\right)+h_{\mathrm{GR}}mg\sin\varphi_{P}\left(k\right)-T_{f}\left(k\right)-T_{r}\left(k\right)\\ -2\left(s_{S,f}^{2}c_{S,f}+s_{S,r}^{2}c_{S,r}\right)\sin\varphi_{P}\left(k\right)\\ -\left(\left(d_{\mathrm{fl}}\left(k\right)+d_{\mathrm{fr}}\left(k\right)\right)s_{D,f}^{2}\right){\dot{\varphi }}_{P}\left(k\right)\cos\varphi_{P}\left(k\right)\\ -\left(\left(d_{\mathrm{rl}}\left(k\right)+d_{\mathrm{rr}}\left(k\right)\right)s_{D,r}^{2}\right){\dot{\varphi }}_{P}\left(k\right)\cos\varphi_{P}\left(k\right)=J_{xx}\;{\ddot{\varphi }}_{P}\left(k\right) \end{array}$$

$T_{f}$ and $T_{r}$ present the counter roll torques at the front and rear axle, respectively. Moreover, $d_{\mathrm{fl}}$, $d_{\mathrm{fr}}$, $d_{\mathrm{rl}}$ and $d_{\mathrm{rr}}$ denote the damping factors of the semi-active dampers located at the suspension front left, front right, rear left and rear right, respectively. The gravitational acceleration is represented by g. The springs are characterized by the spring stiffnesses $c_{S,i}$ and the lever arms $s_{S,i}$ from force application points to the vehicle center plane. The index i indicates the respective vehicle axle. The lever arms of the semi-active dampers are denoted by $s_{D,i}$. The moment of intertia of the vehicle body around the longitudinal axis is specified by $J_{xx}$ and the distance between the center of gravity and the roll center by $h_{\mathrm{GR}}$. 

By physically describing the roll behavior, the roll acceleration ${\ddot{\varphi }}_{P}\left(k\right)$ at time k can be determined using Equation (3). Integrating the roll acceleration ${\ddot{\varphi }}_{P}\left(k\right)$ twice using the explicit Euler method [23], the roll rate ${\dot{\varphi }}_{P}\left(k+1\right)$ and roll angle $\varphi_{P}\left(k+1\right)$ are determined:

(4) $${\dot{\varphi }}_{P}\left(k+1\right)={\dot{\varphi }}_{P}\left(k\right)+{\ddot{\varphi }}_{P}\left(k\right)t_{S},$$

(5) $$\varphi_{P}\left(k+1\right)=\varphi_{P}\left(k\right)+{\dot{\varphi }}_{P}\left(k\right)t_{S}.$$

$t_{S}$ denotes the fixed step sizes of the state estimation, which equals 0.001 s. The only measured input variable is therefore the lateral acceleration $a_{y}$:

(6) $$s_{V,P}=a_{y}.$$

### 3.4. Unscented Kalman Filter

The final step of the hybrid method is to combine the estimation by the artificial neural network with the estimation by the physical model as a function of the confidence level $\tau_{\mathrm{HSE}}$. An unscented Kalman filter implements this combination. The basic functionality of the Kalman filter remains unchanged [24]. The estimation by the artificial neural network is considered here as a measurement.

In order to perform the combination of the two individual virtual sensors depending on the confidence level $\tau_{\mathrm{HSE}}$, the covariances of the transition $Q_{\mathrm{HSE}}$ and measurement $R_{\mathrm{HSE}}$ are manipulated:

(7) $$Q_{\mathrm{HSE}}=\tau_{\mathrm{HSE}}Q_{N},$$

(8) $$R_{\mathrm{HSE}}=\left(1-\tau_{\mathrm{HSE}}\right)R_{N}.$$

The covariances $Q_{\mathrm{HSE}}$ and $R_{\mathrm{HSE}}$ are thus functions of the confidence level $\tau_{\mathrm{HSE}}$. Furthermore, $Q_{N}$ and $R_{N}$ are fixed neutral covariances that are defined before using the hybrid method. 

This definition of the covariances implies that at a confidence level of $\tau_{\mathrm{HSE}}=0$, the resulting estimation by the unscented Kalman filter $x_{\mathrm{HSE}}$ is completely based on the physical model. As the confidence level increases, the estimation of the artificial neural network $x_{\mathrm{ANN}}$ is increasingly taken into account [16].

## 4. Results

In the following, the hybrid method of state estimation is validated in terms of its reliability. It is used in the closed-loop simulation, which is shown in Figure 1.

For the validation, one driving maneuver from the test dataset of [16] is employed. The driving maneuver used is based on steady-state circular driving according to [25]. Thereby, the steady-state behavior during cornering can be described. The velocity of the vehicle is 90 km/h and the cornering radius equals 100 m. Furthermore, there is no lateral road gradient. The steering direction is clockwise. 

In order to validate the reliable operation of the hybrid method, three situations are considered in the following, which would have fatal consequences for the safety of the vehicle if the artificial neural network was not safeguarded. To generate these situations, the input signal of the steering wheel angle δ is manipulated by a drift, an offset and a complete failure, respectively. In addition to this test-driving maneuver, a lap on the Hockenheimring racetrack is used to assess the hybrid method in a holistic way.

### 4.1. Sensor Drift

**Figure 4:**
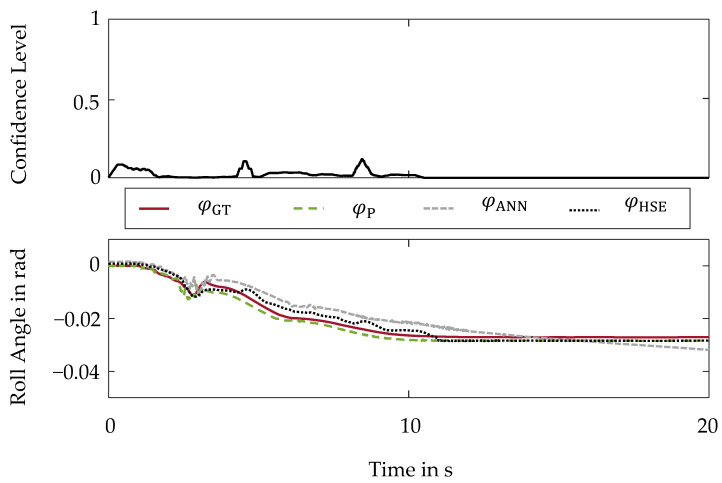
Hybrid Method of State Estimation-Sensor Drift: (**Top**): Confidence Level; (**Bottom**): Roll Angle Curves.

Within the first validation scenario, the behavior of the hybrid method is examined for the manipulation by a sensor drift [26]. The signal of the steering wheel angle δ, an input signal into the artificial neural network, is manipulated by a drift of 3.5°/s from second 10 onward. To evaluate the reliability, the roll angle curves are shown in the bottom part of Figure 4.

Here, a black dotted line represents the hybrid state estimation $\varphi_{\mathrm{HSE}}$. In addition, the individual virtual sensors based on the artificial neural network and the physical model are displayed. A green dashed line represents the estimation by the physical model $\varphi_{P}$ and a gray fine dashed line the estimation by the artificial neural network $\varphi_{\mathrm{ANN}}$. A red line further illustrates the ground truth roll angle $\varphi_{\mathrm{GT}}$.

The influence of the sensor drift in the input signal of the artificial neural network on the estimation $\varphi_{\mathrm{ANN}}$ becomes evident by also observing a drift in the estimation $\varphi_{\mathrm{ANN}}$. Without any additional safeguarding of the artificial neural network, this sensor malfunction would result in an incorrect basis for the control and thus would compromise the safety of the vehicle. However, by using the hybrid method, the sensor malfunction is detected and reliably intercepted.

After the detection of the sensor drift, there is no more confidence in the artificial neural network, which is implemented by a confidence level of $\tau_{\mathrm{HSE}}=0$. The course of the confidence level $\tau_{\mathrm{HSE}}$ is shown in the top part of Figure 4.

At a confidence level of $\tau_{\mathrm{HSE}}=0$, the hybrid method is completely based on the reliable physical model, which is unaffected by the sensor drift due to redundant input variables.

### 4.2. Sensor Offset

**Figure 5:**
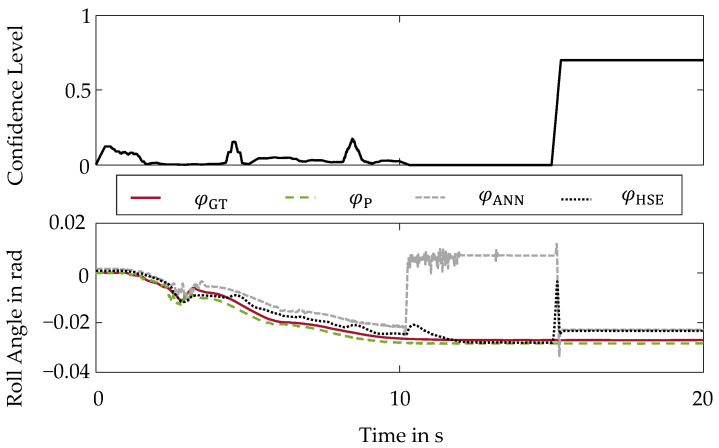
Hybrid Method of State Estimation-Sensor Offset: (**Top**): Confidence Level; (**Bottom**): Roll Angle Curves.

The second validation scenario evaluates the behavior of the hybrid state estimation method in the presence of another typical sensor error which is an offset [27]. Analogous to the sensor drift, the measurement signal of the steering wheel angle δ is also manipulated for the sensor offset. For this purpose, an offset of 165° is applied to the steering wheel angle signal in the period from 10 to 15 s. To evaluate the impact of the sensor offset, the resulting roll angle curves are shown in the bottom part of Figure 5. 

The color scheme remains consistent with Figure 4. The offset in the steering wheel angle signal also creates an offset in the roll angle estimation $\varphi_{\mathrm{ANN}}$ by the artificial neural network. This results in a positive roll angle estimation $\varphi_{\mathrm{ANN}}$ between 10 and 15 s, although a negative ground truth roll angle $\varphi_{\mathrm{GT}}$ is present. The offset is correctly detected by the hybrid method of state estimation. The confidence level equals $\tau_{\mathrm{HSE}}=0$ in this case. Thus, the incorrect estimation $\varphi_{\mathrm{ANN}}$ by the artificial neural network is intercepted and a valid and reliable state estimation $\varphi_{\mathrm{HSE}}$ is provided, which does not compromise the safety of the vehicle.

### 4.3. Sensor Failure

**Figure 6:**
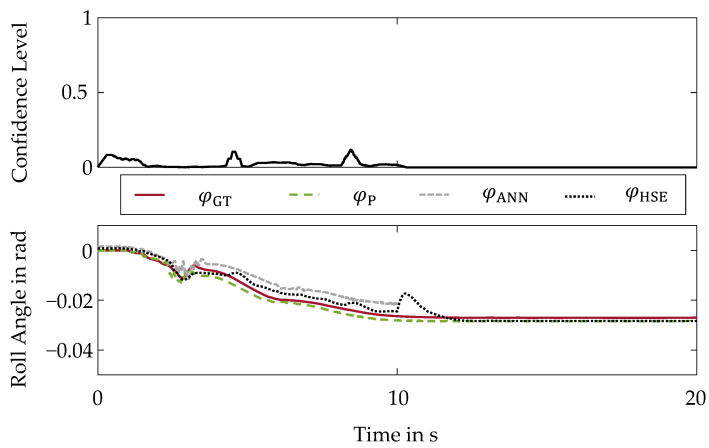
Hybrid Method of State Estimation-Sensor Failure: (**Top**): Confidence Level; (**Bottom**): Roll Angle Curves.

Within the third validation scenario, a failure of the steering wheel angle sensor is set up. The sensor failure occurs at second 10. After the failure, the steering wheel angle signal is no longer available. Thus, the artificial neural network is no longer capable of estimating the roll angle. The resulting roll angle curves are illustrated in the bottom part of Figure 6.

The signal failure is detected successfully by the hybrid method. The confidence level $\tau_{\mathrm{HSE}}$ shown in the top part of Figure 6 constantly adopts a value of $\tau_{\mathrm{HSE}}=0$ after the failure. This is equivalent to no confidence in the artificial neural network. From the sensor failure onward, the hybrid state estimation $\varphi_{\mathrm{HSE}}$ is thus completely based on the physical model $\varphi_{P}$.

By using the hybrid method and the redundant input variables, even the critical case of a sensor failure can be safeguarded. The central predictive vehicle dynamics control system therefore relies on a valid and reliable state estimation $\varphi_{\mathrm{HSE}}$ for this critical scenario as well and can perform its tasks unaffected.

### 4.4. Hockenheimring Racetrack

**Figure 7:**
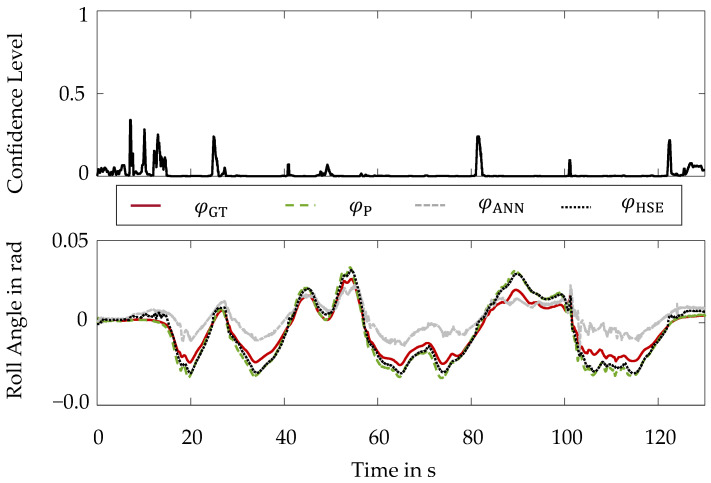
Hybrid Method of State Estimation-Hockenheimring Racetrack: (**Top**): Confidence Level; (**Bottom**): Roll Angle Curves.

The performance of the hybrid method of state estimation is additionally evaluated for a lap on the Hockenheimring racetrack. For this purpose, the roll angle courses resulting from the individual virtual sensors are plotted in the bottom part of Figure 7. 

Moreover, the course of the confidence level during the lap on the Hockenheimring is illustrated in the top part of Figure 7.

As can be observed from the confidence level, the vehicle dynamic states present are unknown to the artificial neural network for a majority of the time. Basically, the performance of the physical virtual sensor is superior to that of the artificial neural network. Nevertheless, there are also regions where the performance of the artificial neural network is superior to that of the physical model. Therefore, by combining the two models within the hybrid method, an improvement of the estimation quality is achieved compared to both individual models.

**Figure 8:**
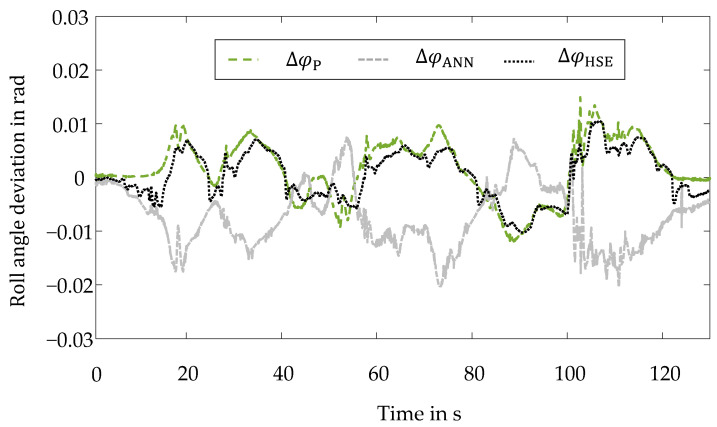
Hybrid Method of State Estimation-Hockenheimring Racetrack: Roll Angle Deviation Curves.

This conclusion is confirmed by examining the deviation of the individual virtual sensors from the ground truth roll angle $\varphi_{\mathrm{GT}}$. These deviations are shown in Figure 8.

To confirm this subjective impression quantitatively, the deviation of the individual virtual sensors from the ground truth is determined by the root mean squared error. The artificial neural network exhibits a larger root mean squared error of 0.0093 rad than the physical model with a root mean squared error of 0.0047 rad. By using the hybrid method, the estimation quality can be improved by 50% compared to the artificial neural network and about 16% compared to the physical model. Here, a root mean squared error of 0.0047 rad is present. Table 3 summarizes the root mean squared errors.

## 5. Conclusions

This contribution focuses on safeguarding the reliability of artificial neural networks employed for the task of state estimation. The application example is the roll angle estimation of a vehicle. Due to the black-box characteristic of the artificial neural network, the reliability cannot be guaranteed throughout. For this purpose, the hybrid state estimation method is presented, which safeguards the artificial neural network by a fully comprehensible and reliable physical model. The implementation and validation is based on a simulation framework comprising IPG CarMaker and MATLAB/Simulink. Within this simulation framework, the state estimation is in a closed loop with a central predictive vehicle dynamics control, actuator models and the vehicle itself. Thus, an invalid or unreliable state estimate would directly affect the vehicle safety. For validation, three aggravated scenarios are set up that directly affect the artificial neural network by manipulating an input signal. These scenarios include a sensor drift, a sensor offset, and a sensor failure, respectively. In all scenarios, the estimation by the artificial neural network is distorted, which would bear fatal consequences in combination with the central predictive control. All sensor malfunctions are detected and handled successfully by the hybrid method, resulting in a valid and reliable estimate besides the malfunctions. In addition, the performance of the hybrid method is evaluated for a lap on the Hockenheimring racetrack. Ultimately, the vehicle safety is maintained and the estimation accuracy is improved. Future work will focus on the generalizability of the proposed hybrid method.

## Figures and Tables

**Table 1:** Hyperparameter Optimization—Fixed Parameters.

Parameter	Value
Batch Size	1024
Evaluation Metric	Mean Absolute Error
Number of Epochs	50
Optimizer	Adam
Type of Layers	Long Short-Term Memory

**Table 2:** Artificial Neural Network—Hyperparameters.

Hyperparameter	Value
Number of Recurrent Layers	4
Number of Neurons within one Recurrent Layer	129
Regularization	None
Temporal Lookback	4

**Table 3:** Estimation Quality Validation.

Virtual Sensor	Root Mean Squared Error
Artificial Neural Network	0.0093 rad
Hybrid Method	0.0047 rad
Physical Model	0.0056 rad

## Data Availability

Not applicable.
